# Factors Associated with Shelter Dog Euthanasia versus Live Release by Adoption or Transfer in the United States

**DOI:** 10.3390/ani11040927

**Published:** 2021-03-25

**Authors:** Cassie J. Cain, Kimberly A. Woodruff, David R. Smith

**Affiliations:** 1 Department of Pathobiology and Population Medicine, Mississippi State University College of Veterinary Medicine, Mississippi State, MS 39762, USA; cjc595@msstate.edu (C.J.C.); DSmith@cvm.msstate.edu (D.R.S.); 2 Department of Clinical Sciences, Mississippi State University College of Veterinary Medicine, Mississippi State, MS 39762, USA

**Keywords:** animal shelter, shelter dogs, euthanasia, live release, United States

## Abstract

**Simple Summary:**

United States animal shelters care for unwanted dogs until they are adopted, transferred to another facility, or euthanized. Previous studies have determined that certain phenotypic characteristics can be used to predict the outcome of shelter dogs. However, these earlier studies have typically been limited by sample size, shelter geographic location, and/or the number of shelters participating in the study, thus reducing generalized applicability of the results. The aim of this study was to test if certain characteristics of dogs in shelters predict the decision for those dogs to be euthanized rather than experience a live release by adoption or transfer. This study may be valuable to shelter staff because utilizing such phenotypic information can help shelter employees focus adoptability protocols, such as socialization and training programs, on dogs with a greater chance of being euthanized.

**Abstract:**

The objective of this study was to identify phenotypic characteristics predicting the outcome of euthanasia for dogs entering shelters compared to live release. Individual dog records for 2017 were requested from shelters in five states (Mississippi, Pennsylvania, Michigan, Colorado, and Oklahoma) receiving municipal funding and using electronic records. Duplicate dogs were removed and records from 17 shelters were merged into a dataset of 25,047 unique dogs with variables of breed, gender, coat color, size, age, region, and time in shelter. Only data from dogs with the potential to be adopted (*n* = 19,514) were analyzed. From these data, a simple random sample of 6200 dogs was used for modelling. Variables describing coat length, estimated adult size, and skull type were imputed from the breed description. A Cox proportional hazard model with a random effect of shelter was developed for the outcome of euthanasia using manual forward variable selection and significance for variable retention at alpha = 0.05. A size by geographic region interaction was associated with the hazard of euthanasia (*p* = 0.0204). Additionally, age group and skull type were both associated with euthanasia compared to live release (*p* < 0.0001). The results of this study indicate that phenotypic characteristics of dogs are predictive of their hazard for euthanasia in shelters.

## 1. Introduction

Over the years, awareness for shelter dog euthanasia has increased among the US general public and veterinarians alike. In 1988, an estimated 9.9 to 16.6 million dogs were euthanized in shelters [1]. However, these estimates were often limited by a lack of sufficient evidence regarding the number of shelters present in the United States and, subsequently, the number of dogs in shelters’ care [2]. Currently, it is estimated that 670,000–777,000 shelter dogs are euthanized each year [3,4]. These estimates are markedly decreased from previous estimates, suggesting that intervention programs, such as an increase in pet sterilization or transfer programs, have successfully increased the probability of a dog’s live release from shelters. Live release describes successful outcomes for shelter dogs, including adoption, return to owner, or transfer, compared to the unsuccessful outcome of euthanasia.

Although the number of shelter dogs euthanized has been decreasing, the option remains for shelters to euthanize dogs if they believe such dogs are unlikely to be rehomed. Although this is a difficult decision, identifying phenotypic traits or characteristics of the dogs that are associated with less favorable outcomes may help shelter staff make more objective decisions about allocation of resources. Previous studies have demonstrated that factors such as age, size, and geographic location all with an interaction of length of shelter stay, all affect shelter dog adoption [5].

Additional phenotypes and characteristics such as age group and coat color have also been found to affect shelter dog euthanasia. Several researchers have identified black or dark coated dogs as having an increased risk of euthanasia [6,7,8]. Another study asked potential adopters to rank photographs of shelter dogs from most attractive to least attractive. They found that dogs which were eventually adopted were ranked as the most attractive, and the ones eventually euthanized were ranked as least attractive [9].

This evidence suggests that phenotype is associated with euthanasia in shelter dogs. However, studies identifying phenotypic traits associated with euthanasia are typically limited by the number of shelters used in analysis, the shelter’s geographic location, or the sample size of dogs studied. With such limitations in mind, the objective of this study was to determine factors associated with shelter dog euthanasia, compared to adoption or transfer outcomes of shelter dogs using a representative sample of dogs from five US states.

## 2. Materials and Methods

The complete materials and methods for this study have been described previously [5]. Briefly, shelters were chosen for inclusion in this study from a previously compiled census of shelters in five study states: Mississippi, Pennsylvania, Michigan, Colorado, and Oklahoma. This list represented 342 shelters. Only 86 shelters that received municipal funding and kept electronic records were included in the final shelter frame because municipally funded shelters were those shelters most likely to be open-admission and electronic records were necessary to facilitate data collection. A total of 17 of 86 (20%) shelters provided records for this study.

The subjects of interest in this study were dogs with the potential for adoption or transfer. Therefore, dogs with an outcome of owner requested euthanasia, returned to owner, or deceased on arrival were not included in the final dataset. Dogs with a length of stay (LOS) equal to zero days were also excluded from analysis. The final dataset contained 19,514 unique dogs.

### 2.1. Dataset Variables

Due to the common misidentification of dog breed, phenotypic traits created by searching public pet adoption websites for common breed characteristics were imputed from each primary breed estimate, including predicted adult size, coat length, and skull type.

Skull type was categorized as brachycephalic (e.g., Pugs, bulldogs); dolichocephalic, characterized by long heads and noses (e.g., hounds, collies); and mesocephalic skull type, which have heads that are a fair medium between the two extremes (e.g., Labradors, cocker spaniels). Guidelines to aid in assigning skull types to each breed were used to decrease misclassification [10,11]. A blockhead variable was imputed from primary and secondary breeds to identify dogs that characteristically have square-shaped heads. If dogs were described as pit bulls, Staffordshire terriers, boxers, Cane Corsos, mastiffs, English Bulldogs, bulldogs, American Bulldogs, or rottweilers, they were considered to be blockheaded [12].

Breeds were categorized by body weight as “small,” “medium,” “large,” and “giant” if their expected adult weights were less than or equal to 13.6 kg, greater than 13.6 kg to less than or equal to 22.7 kg, greater than 22.7 kg to less than or equal to 31.8 kg, or greater than 31.8 kg, respectively. If dogs were puppies, the size entry was changed to reflect breed estimated adult size. As giant dogs only represented 2% of entries in the dataset, the giant category was collapsed into the large category. The coat length variable was assigned as either short, medium, or long. As medium coated dogs included only 10% of entries, the medium coat length group was collapsed into the long coat length group.

Primary and secondary coat colors were categorized into 8 colors (black, brown, red, grey, white, tan, yellow, and blue) from 44 different variants of color reported in the records. 

Age group was categorized as “puppies,” “young adults,” “adults,” and “seniors” if the reported age was less than or equal to 6 months, greater than 6 months to 2 years, greater than 2 years to less than 8 years, or greater than or equal to 8 years, respectively. As there is no phenotypic indicator to differentiate the age of young adults versus adults, as there is for puppies at approximately 6 months with the eruption of permanent canines, the young adult group was combined with the adult group.

The geographical region of each shelter was categorized as southern, northern, or western. The southern region included shelters from Mississippi and Oklahoma. The northern region included shelters from Michigan and Pennsylvania. The western region included shelters from Colorado.

### 2.2. Data Analysis

Inferential statistics were computed using SAS for Windows v9.4 (SAS Institute, Inc., Cary, NC, USA), and sample size calculations were performed using Epi Info (CDC, Atlanta, GA, USA). Crude descriptive statistics were completed using spreadsheet software (Excel v16, Microsoft, Redmond, WA, USA).

An extended Cox proportional hazard regression model was created through manual forward variable selection. Variables were retained in the model if Wald type 3 *p*-values were significant (alpha = 0.05). Shelter was included as a random effect in the model. Age group, coat length, estimated adult size, skull type, presence of a blockhead, region, primary coat color, and gender were tested as fixed effects. To improve model stability, the length of stay was limited to 80 days, after which a dog was considered censored.

To reduce the ability to detect very small differences in independent variables, a simple random sample was taken from the dataset of 19,514 potentially adoptable dogs using SAS, PROC SURVEY SELECT. Using the cohort study sample size calculator from Epi Info, a sample size of approximately 6200 dogs was determined to be sufficient to detect a risk ratio of 1.6, at a 95% confidence level, assuming 95% power.

The proportional hazard assumption was tested by creating and testing a time interaction variable for each fixed effect. Variables with time interactions were depicted graphically using methods described by Dohoo [13]. Multiple comparisons were adjusted using Tukey–Kramer methods. Dogs with incomplete information for all of the phenotypes included in the model were ultimately excluded.

## 3. Results

Of the dogs with the potential to be adopted or transferred, the probability of a dog to be euthanized was 14%. The median LOS for euthanized dogs was 6 days with a mean (standard deviation) of 9 days (10.7 days). The frequency of dogs with each phenotype tested for inclusion in the model are summarized in Table 1.

Age group was associated with the hazard for euthanasia of shelter dogs (*p* < 0.0001) and met the proportionality assumption. Puppies were less likely to be euthanized compared to adults (HR = 0.42, 95% C.I. 0.30–0.58). Puppies were also less likely to be euthanized compared to seniors (HR = 0.16, 95% C.I. 0.11–0.24). Adult dogs were less likely to be euthanized compared to seniors (HR = 0.39, 95% C.I. 0.30–0.51).

Skull type was found to be a factor influencing euthanasia of shelter dogs (*p* < 0.0001) and met the proportionality assumption. Brachycephalic dogs were more likely to be euthanized when compared to mesocephalic dogs (HR = 1.88, 95% C.I. 1.50–2.34), and brachycephalic dogs were also more likely to be euthanized when compared to dolichocephalic dogs (HR = 2.21, 95% C.I. 1.63–3.00). Mesocephalic dogs compared to dolichocephalic dogs had no difference in euthanasia risk (*p* = 0.45). 

**Figure 1:**
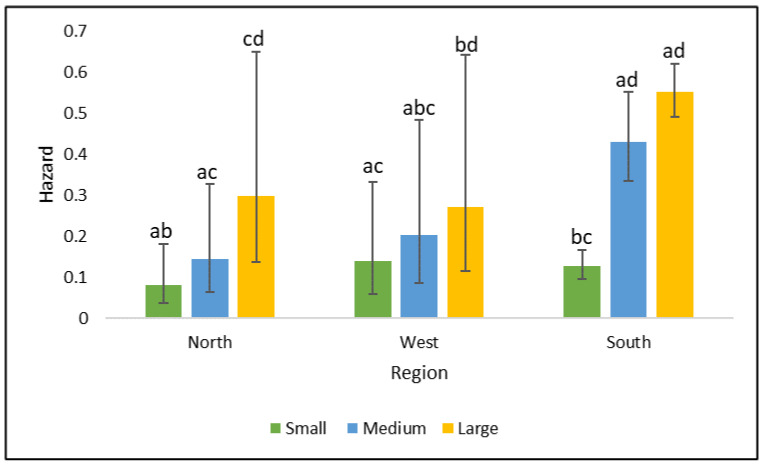
Euthanasia model adjusted region by size interaction displayed as hazards, estimated from the simple random sample of 4500 dogs. Error bars display one standard error from the estimated hazard ratios. Labels a–d indicate differences among hazards. Adjustment for multiple comparisons was performed using Tukey–Kramer methods.

An interaction between region and mature size was also found to influence shelter dog euthanasia (*p* = 0.0204) as displayed in Figure 1. This interaction demonstrates that large dogs had a greater hazard for euthanasia than small dogs in every region. The south also demonstrated a greater hazard for euthanasia of medium dogs than small dogs. Adjustment for multiple comparisons was performed using Tukey–Kramer methods. Variables included in the final model with adjusted HR estimates can be found in Table 2.

## 4. Discussion

Certain dog phenotypes, including age group, skull type, region, and size, were all associated with an increased hazard for euthanasia. All of the phenotypes associated with euthanasia had constant hazards for euthanasia over time. This suggests that, although LOS was short, it did not influence shelter employees’ decisions to euthanize dogs in their care; rather, phenotype was a better predictor of which dogs were euthanized.

This study identified an association between age group and euthanasia. Puppies were identified as having the greatest chance of live release compared to both adults and seniors. Other studies have found that puppies are the most appealing group to be adopted and seniors are the least appealing group for adoption [14]. Shelters may be less willing to euthanize puppies in their care unless additional circumstances arise, such as a disease outbreak. Previous studies suggest that senior dogs older than 9 years have the longest LOS [15], seniors have half the odds of live release compared to puppies [12], and that seniors between the age of 10 and 12 years old are more likely to be euthanized [16]. Shelters may be more willing to euthanize seniors because they may be more likely to have chronic health problems, lower adoption rates, or persistent behavioral concerns.

This study also associated skull type with an increased hazard for shelter dog euthanasia. Brachycephalic dogs were found to be at a greater risk for euthanasia than both mesocephalic and dolichocephalic dogs. To the author’s knowledge, this is the first study to fully identify the effect of skull type on shelter dog euthanasia, although previous research has described increased euthanasia associated with blockheaded dogs. Blockheaded dogs are typically brachycephalic so these results are consistent with the findings of a previous study that the presence of a “blockhead,” or a square-shaped head, reduces shelter dog’s chances of live release [12].

The breed or breed variations with a brachycephalic skull type include pit bulls, Staffordshire terriers, or American Bulldogs. Previous studies have found that these breeds are often less likely to have favorable outcomes or live release [16,17,18,19,20]. Although the practice of determining euthanasia risk based off of breed phenotype or breed grouping is common, shelter personnel mislabel breeds when dogs enter the shelter 5–25% of the time [20,21]. Additionally, shelter employees may purposefully mislabel breed if breed-specific legislation is present in the community [22]. Therefore, analyzing on breed or breed group may not be the best factor to determine the risk for euthanasia.

Another interesting finding from this study was the identification of a region by size interaction. Previous studies have shown that larger dogs are more likely to be euthanized [7], and that some regions are more likely to euthanize larger dogs than others [4]. However, no study has described an interaction between size and region. These results show that within each region, small dogs compared to large dogs are less likely to be euthanized and, in the south, small dogs are also less likely to be euthanized than medium-sized dogs. This region–size interaction is important because it demonstrates that large dogs are more likely to be euthanized compared to small dogs, despite their location, but that medium-sized dogs are also at more of a risk in certain regions. This interaction may be explained by a higher chance of adoption of small breed dogs, or by an increased probability to transfer small-sized dogs. Large dogs may be at a greater risk for euthanasia because of their perceived requirement for more space or exercise, which is increasingly more difficult for pet-owners to provide due to working hours and living space. As 80% of the US human population lives in urban areas [23], this may help explain why large shelter dogs have an increase in euthanasia risk. Large dogs may also be less likely to be included in transfer programs because of space requirements during transport.

Misclassification bias may have been introduced into this study when imputing phenotypes from breed. For example, when estimated adult size, skull type, and coat length were imputed from the listed breed, some dogs had a breed listing of “mixed breed” or “terrier.” These breed designations did not allow for phenotypes to be imputed and, thus, those categories were left blank. However, the misclassification is likely non-differential resulting in a conservative bias towards the null hypothesis.

The results from this study may indicate the preferences of people who adopt dogs from shelters, or they may be a reflection of shelter staff’s preconceived notions about which dogs are adoptable, which may guide euthanasia decisions. Regardless of who is making the decision (for adoption or euthanasia), the results of this study explain and help predict the relationship between a dog’s physical attributes and its risk for being euthanized.

## 5. Conclusions

This study found that skull type, size, regional location, and age group all have an association with the hazard for shelter dog euthanasia. These results likely reflect the opinions of dog adopters about which dogs are less desirable for adoption in combination with the opinions of shelter workers about which dogs are least adoptable. However, the information identified in this study can help shelter employees make informed, evidence based outcome decisions regarding dogs in their care.

## Figures and Tables

**Table 1:** Frequencies of phenotypes tested for multivariate euthanasia model inclusion using the full dataset 19,514 shelter dogs and the simple random sample (SRS) of 6200 dogs.

Variable	Response	Counts	Frequency (%)	Observations	SRS Counts	SRS Frequency (%)	SRS Observations
Coat length	Short	13,214	69	19,287	4232	69	6130
	Long	6073	31		1898	31	
Skull type	Brachycephalic	5875	32	18,648	1888	32	5921
	Mesocephalic	9162	49		2900	49	
	Dolichocephalic	3611	19		1133	19	
Estimated adult size	Small	5503	28	19,356	1715	28	6150
	Medium	4290	22		1361	22	
	Large	9563	49		3074	50	
Blockhead type	Present	4163	21	19,514	1333	22	6200
	Not Present	15,351	79		4867	78	
Coat color	Black	5920	37	16,150	1886	37	5115
	Blue	364	2		103	2	
	Brown	2147	13		668	13	
	Grey	490	3		139	3	
	Red	1064	7		315	6	
	Tan	3304	20		1091	21	
	White	2539	16		812	16	
	Yellow	322	2		101	2	
Gender	Male	10,020	52	19,302	3171	52	6121
	Female	9258	48		2950	48	
Age group	Puppy	4026	22	18,605	1285	22	5916
	Adult	12,973	69		4114	69	
	Senior	1606	9		517	9	
Region	South	3948	20	19,514	1249	20	6200
	North	7168	37		2298	37	
	West	8398	43		2653	43	

**Table 2:** Extended Cox regression model for euthanasia using the simple random sample of 6200 dogs. Adjustment for multiple comparisons was performed using Tukey–Kramer methods.

Phenotype	Parameter Estimate	Standard Error	Adj *p*-Value	Hazard Ratio	Adj Lower Confidence Limit	Adj Upper Confidence Limit
Puppies vs. adults	−0.871	0.142	<0.0001	0.418	0.300	0.583
Puppies vs. seniors	−1.807	0.170	<0.0001	0.164	0.110	0.245
Adults vs. seniors	−0.934	0.113	<0.0001	0.392	0.301	0.511
Brachycephalic vs. mesocephalic	0.630	0.095	<0.0001	1.877	1.503	2.342
Brachycephalic vs. dolichocephalic	0.792	0.131	<0.0001	2.207	1.625	2.997
Mesocephalic vs. dolichocephalic	0.162	0.135	0.4535	1.176	0.857	1.614
North, small vs. medium	−0.567	0.368	0.593	0.567	0.393	0.819
North, small vs. large	−1.305	0.226	<0.0001	0.271	0.216	0.340
North, medium vs. large	−0.737	0.316	0.137	0.478	0.349	0.656
West, small vs. medium	−0.382	0.181	0.230	0.683	0.569	0.818
West, small vs. large	−0.672	0.167	0.0004	0.511	0.432	0.604
West, medium vs. large	−0.290	0.155	0.360	0.748	0.641	0.873
South, small vs. medium	−1.221	0.300	0.0004	0.295	0.218	0.398
South, small vs. large	−1.470	0.239	<0.0001	0.230	0.181	0.292
South, medium vs. large	−0.249	0.224	0.870	0.780	0.623	0.975
